# Immune niches for hair follicle development and homeostasis

**DOI:** 10.3389/fphys.2024.1397067

**Published:** 2024-04-22

**Authors:** Artem Kiselev, Sangbum Park

**Affiliations:** ^1^ Institute for Quantitative Health Science and Engineering (IQ), Michigan State University, East Lansing, MI, United States; ^2^ Division of Dermatology, Department of Medicine, College of Human Medicine, Michigan State University, East Lansing, MI, United States; ^3^ Department of Pharmacology and Toxicology, College of Human Medicine, Michigan State University, East Lansing, MI, United States

**Keywords:** hair follicle, development, homeostasis, immune niche, immune privilege, alopecia

## Abstract

The hair follicle is a dynamic mini-organ that has specialized cycles and architectures with diverse cell types to form hairs. Previous studies for several decades have investigated morphogenesis and signaling pathways during embryonic development and adult hair cycles in both mouse and human skin. In particular, hair follicle stem cells and mesenchymal niches received major attention as key players, and their roles and interactions were heavily revealed. Although resident and circulating immune cells affect cellular function and interactions in the skin, research on immune cells has mainly received attention on diseases rather than development or homeostasis. Recently, many studies have suggested the functional roles of diverse immune cells as a niche for hair follicles. Here, we will review recent findings about immune niches for hair follicles and provide insight into mechanisms of hair growth and diseases.

## Correlation between developing hair follicles and immune cells

**FIGURE 1:**
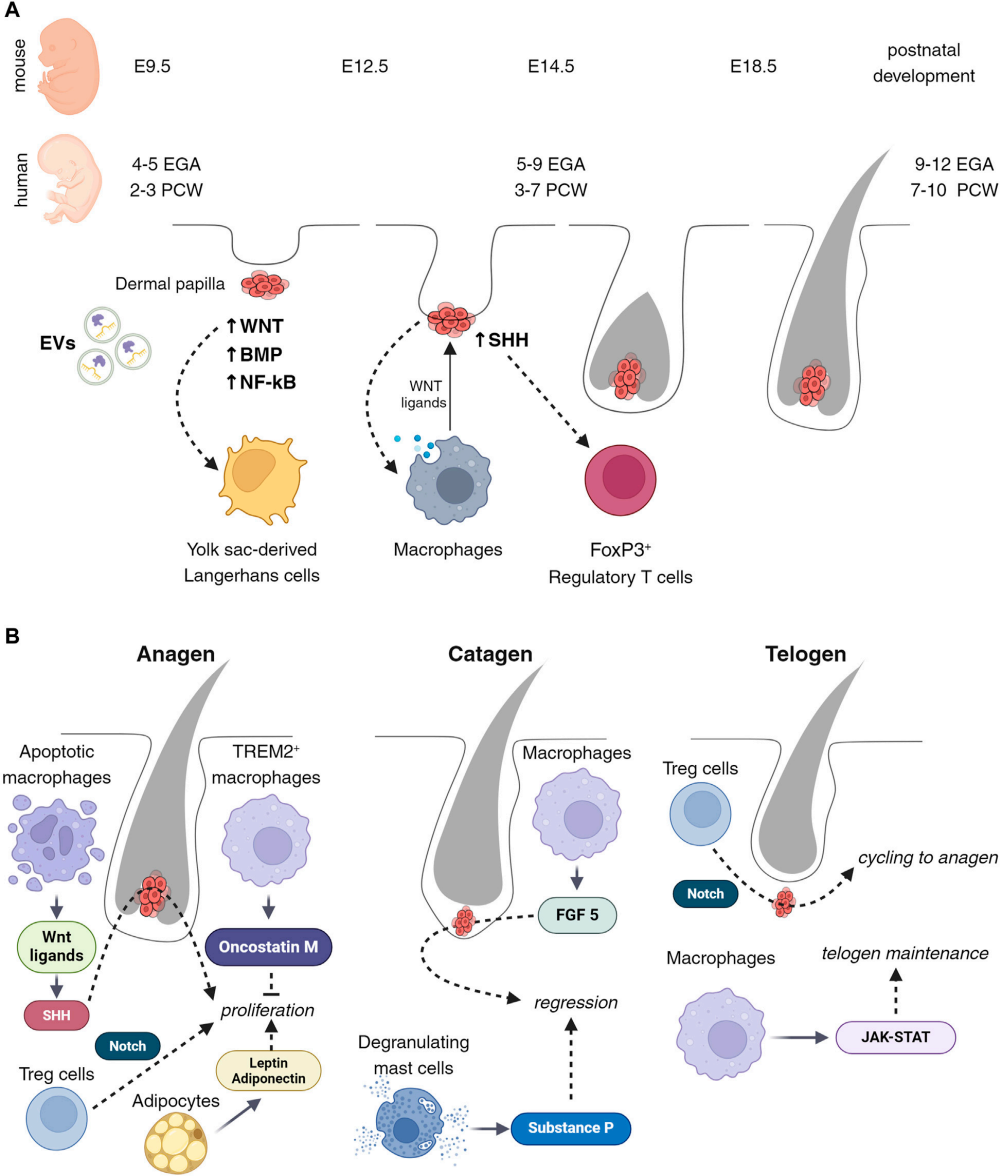
Cell composition of developing and cycling hair follicles. **(A)** Wnt signaling attracts the initial immune cells present in the early stages of placode development. These cells are the atypical yolk sac (YS)-derived Langerhans cells, observable between embryonic days 9.5 and 12.5 or 2 to 3 post-conception weeks (PCW). Subsequently, from embryonic day 12.5–14.5 or 3 to 7 PCW, macrophages emerge, facilitating active morphogenesis, tissue remodeling, and exerting influence on the stem cells of hair follicles via Wnt ligands. Concurrently, regulatory T (Treg) cells appear, with their state seemingly regulated by Sonic Hedgehog (Shh) signaling. **(B)** Throughout the hair follicle cycle, macrophages play a pivotal role in modulating different phases. During the anagen phase, macrophages promote proliferation through the releasing of Wnt ligands followed by activation of Shh, and secretion of Oncostatin M. These factors collectively stimulate the hair follicle cells, leading to their active division and growth. Conversely, during the catagen phase, macrophages facilitate this process via the release of FGF5, leading to the cessation of hair growth and the initiation of follicle regression. Moreover, macrophages are instrumental in maintaining and regulating the telogen stage, through JAK-STAT signaling pathways. This ensures the follicle’s readiness for the subsequent growth phase. Besides macrophages, adipocytes, T cells, and mast cells also contribute significantly to the hair follicle growth cycle. Through Notch signaling, Treg cells close the hair growth cycle and activate the transition from telogen to anagen.

The development of hair follicles has been well-characterized by several studies (Park, 2022; Wang and Higgins, 2020; Wang et al., 2022; Welle, 2023) (Figure 1A). The first signs of hair placodes can be seen at 4–8 weeks EGA (Estimated gestational age) which is the equivalent of 2-4 PCW (post-conception weeks) in humans and day E9.5-E12.5 in mice. The first signal initiating the process of placode formation is Wnt/β-catenin (Andl et al., 2002), together with NF-κB and BMP (Zhang et al., 2009). Exosomes carrying microRNA miR-181a-5p have recently been proposed as an initiating signal modulating Wnt activity in forming hair placodes (Zhao et al., 2022). Epidermal ablation of Wntless (Wls) or dermal ablation of β-catenin results in the absence of early placode formation (Chen et al., 2012). At the next stage, 5- weeks, EGA human (3–7 PCW), E12.5–14.5 mouse, Sonic-hedgehog signaling becomes activated and the placode develops into hair germs and hair pegs (Chiang et al., 1999; Levy et al., 2005). The hair follicle development in humans occurs mainly *in utero*, but continues after postnatally in mice (Sennett et al., 2015; Saxena et al., 2019; Reynolds et al., 2021; Jacob et al., 2023).

Morphogenesis of hair follicles is mainly focused on crosstalk between epithelial stem cells and mesenchymal niches, such as the dermal papilla. However, skin is composed of diverse cell types and these cells could potentially impact the development of hair follicles. In particular, immune cells appear in the skin from the beginning of the hair follicle formation and are located adjacent to developing hair follicles. In addition, recent studies suggested immune cells directly regulate hair follicle stem cells and can affect the homeostasis of adult skin (Castellana et al., 2014; Ali et al., 2017; Wang et al., 2019). Here, we attempt to review the earliest recorded immune cell possibly influencing embryonic morphogenesis and provide evidence of immune niches for hair follicle stem cells.

### Langerhans cells

Langerhans cells are one of the first immune cells to appear in the developing epidermis from the yolk sac (Hoeffel et al., 2012). At the time of the placode formation (6–12 weeks EGA (4–10 PCW), E12.5), the phenotype and distribution of these cells differ from that observed in adult skin (Foster et al., 1986) (Figure 1A). Epcam, typically expressed in epidermal Langerhans cells, is a direct transcriptional target of the canonical Wnt-β-catenin signaling pathway. At the same time, the Wnt antagonist, Dkk (limiting the area of Wnt activation and promoting placode formation), also affects Langerhans cells and reduces their proliferation (Becker et al., 2011). In adult skin, mechanical stretching also affects the functioning of Langerhans cells by induction of SFRP2/Wnt/beta-catenin (Ledwon et al., 2022). Although Wnt singling affects both LC maturation and hair follicle development, LC depletion did not affect HF formation (Kaplan et al., 2005). This suggests that LCs do not directly regulate hair follicle development.

### Macrophages

Macrophages appear in developing humans at 7 weeks EGA (5 PCW) and mouse skin at E9.5–12.5 (Hoeffel et al., 2015; Kolter et al., 2019; Dhariwala et al., 2020; Reynolds et al., 2021).

Because of the lethal phenotype of macrophage-null mice, the precise functions of these cells during the initiation and early development of hair follicles are not known. However, early fetal macrophages involve clearing debris associated with developmental tissue remodeling and vascularization (Fantin et al., 2010). Macrophages can modulate the Wnt/beta-catenin pathway, either directly through the secretion of Wnt ligands or indirectly by influencing the microenvironment (Malsin et al., 2019) (Figure 1A). A Wnt-mediated connection between macrophages and hair follicle stem cells has been demonstrated (Castellana et al., 2014). At the same time, hair follicle stem cells with activated sonic hedgehog (Shh) and BMP signaling can attract macrophages and influence polarization (Petty et al., 2019; Chakrabarti et al., 2022; Wang et al., 2023). All this suggests that macrophages may play an essential role in the early morphogenesis of hair follicles and provide initiation or regionalization of this process.

### Regulatory T (Treg) cells

Treg cells are observed in developing mouse skin from E6–12 (Scharschmidt et al., 2017), and in humans from the second trimester, 20–23 weeks EGA (18–21 PCW). The density of Treg cells is higher in areas with a higher density of future hair (Dhariwala et al., 2020). The Shh signaling pathway, active during the middle stages of neonatal hair follicle development, has been observed in association with Treg cell activation in a model of atypical dermatitis or in response to mycobacterial infection (Holla et al., 2016; Papaioannou et al., 2019) (Figure 1A). One of the important characteristics of Treg cells in fetal skin is density and the presence of a FOXP3+ memory function (Dhariwala et al., 2020). Recent studies have shown that FOXP3+ CD4^+^ Treg cells are involved in various tissue healing and regeneration by controlling neutrophils and macrophages and local activation of satellite stem cells of the skeletal muscle (Castiglioni et al., 2015; Hou et al., 2021; Goral et al., 2023). These suggest that Treg cells assist the intensive processes of tissue remodeling that occur in the early stages of skin and hair follicle development, regulating homeostasis and tolerance.

Working with human embryos has many limitations including ethical problems. Therefore, many studies suggest mechanisms of human hair follicle development in humans by parallel comparisons with the development in mice. Since the developmental timing of human and mouse hair follicles is different, many comparisons rely on rough morphological similarity (Reynolds et al., 2021). Future studies with advanced single-cell sequencing and spatial transcriptomics technologies will provide accurate and novel cellular and molecular mechanisms of hair follicle development in humans (Rosenberg et al., 2018; Clark et al., 2023; Komatsu et al., 2023).

## Immune niches for homeostasis of hair follicles

One of the special features of hair follicles is the repetitive cyclic phases of growth (anagen), regression (catagen), and rest (telogen) (Schneider et al., 2009; Geyfman et al., 2015; Wang and Higgins, 2020). Evolutionarily, this cycle is necessary to maintain good conditions of hairs in mammals from damage or seasonal changes. Human and mouse hair follicles have the same three phases of cycles but show different progression in timing. In mice, the first two hair cycles are synchronized and are changed as a wave pattern from an anterior to posterior direction. Therefore, it is possible to judge specific hair cycles based on the date of birth. In contrast, humans show different hair cycles depending on the local area because of unsynchronized and mosaic patterns of the hair cycle. In addition, hair follicles in the scalp have a much longer anagen cycle which enables us to make longer hairs compared to mouse hairs (Halloy et al., 2000; Müller-Röver et al., 2001; Ohnemus et al., 2006; Plikus and Chuong, 2008; Purba et al., 2014).

### Anagen

The growth of hair follicles is associated with the activation of Wnt10b, fibroblast growth factor (FGF)-2, and FGF-7, and the suppression of BMP (Li et al., 2011; Hwang et al., 2012). In experiments on mice, it was shown that the release of Wnt ligands is associated with apoptosis of macrophages, which leads to the proliferation of hair germ cells via activation of Shh signaling, in hair follicle stem cells (Castellana et al., 2014; Ali et al., 2017). Additionally, a specialized subset of macrophages, known as TREM2+ trichophages, has been identified to produce oncostatin M, which inhibits hair follicle stem cell proliferation and differentiation, thus fine-tuning the growth phase duration (Oro and Higgins, 2003). At the same time, Treg cells are attracted adjacent to the hair bulge and activate hair follicle stem cells via Notch signaling with the high level of Jagged 1 (Ali et al., 2017). Adipocytes also participate in the precise control and synchronization of the growth phase. Secretion from adipocytes, such as leptin, adiponectin, and growth factors, supports the active proliferation of hair cells (Park et al., 2010; Won et al., 2012; Cruz et al., 2023) (Figure 1B).

### Catagen

Growing hair follicles eventually enter the regression phase. The size of hair follicles dramatically decreased within a few days by apoptosis and extrusion (Elliott et al., 1999; Müller-Röver et al., 2001; Purba et al., 2014). This regression is influenced by signals from the microenvironment, including BMP from adipose tissue and TGF-β from the dermal papilla. The catagen can be regulated by macrophages that secrete FGF-5. In humans, FGF-5 mutations lead to trichomegaly and eyelash loss, as well as retention of hair follicles at the catagen stage (Müller-Röver et al., 2001; Higgins et al., 2014). Depletion of mast cells in mice also showed a delay in entry into catagen (Arck et al., 2005). This is apparently due to the inability of such mice to secrete substance P, which is usually contained in mast cell granules and is released during mast cell degranulation during entry into catagen (Peters et al., 2007) (Figure 1B).

### Telogen

The hair follicles enter a resting phase after the catagen. During this time, both hair follicle stem cells and dermal papilla become quiescent. This quiescence is maintained by inhibitory signals from various niche components, including the Keratin6 +inner bulge cells, arrector pili muscle, subdermal adipocytes, and immune cells (Fujiwara et al., 2011; Hsu et al., 2011; Wang et al., 2019). Immune cells, especially Treg cells and macrophages, vary in number throughout the hair cycle, playing key roles in tissue maintenance. Macrophage and Treg numbers are abundant in early-mid telogen and gradually decrease until the onset of Anagen (Castellana et al., 2014; Ali et al., 2017). Experiments on mice showed macrophage ablation during telogen can trigger the transition to anagen (Castellana et al., 2014; Wang et al., 2019). While Treg cells activate hair follicle stem cells via Notch signaling for initiation of anagen, macrophages maintain telogen by activity of JAK-STAT signaling (Ali et al., 2017; Dalessandri and Kasper, 2019) (Figure 1B).

The elucidation of immune cell roles across different phases of the hair cycle opens promising avenues for therapeutic interventions targeting hair loss and other follicle-related disorders. Future research should aim to further dissect the molecular dialogues between hair follicles and immune cells. In particular, recent advance in technologies in single-cell analyses, such as single-cell RNA sequencing and intravital imaging, offers an opportunity to uncover novel cell types and signaling pathways involved in hair follicle regulation, potentially identifying targets for enhancing hair growth or delaying hair loss. Additionally, the therapeutic manipulation of immune pathways, such as modulating Treg cell activity or macrophage phenotypes, holds potential for novel treatments. Ultimately, integrating these insights with advances in gene editing and regenerative medicine could lead to groundbreaking strategies for hair restoration, emphasizing the importance of a detailed understanding of the hair follicle immune microenvironment.

## Regulation of the immune privilege of hair follicles

**FIGURE 2:**
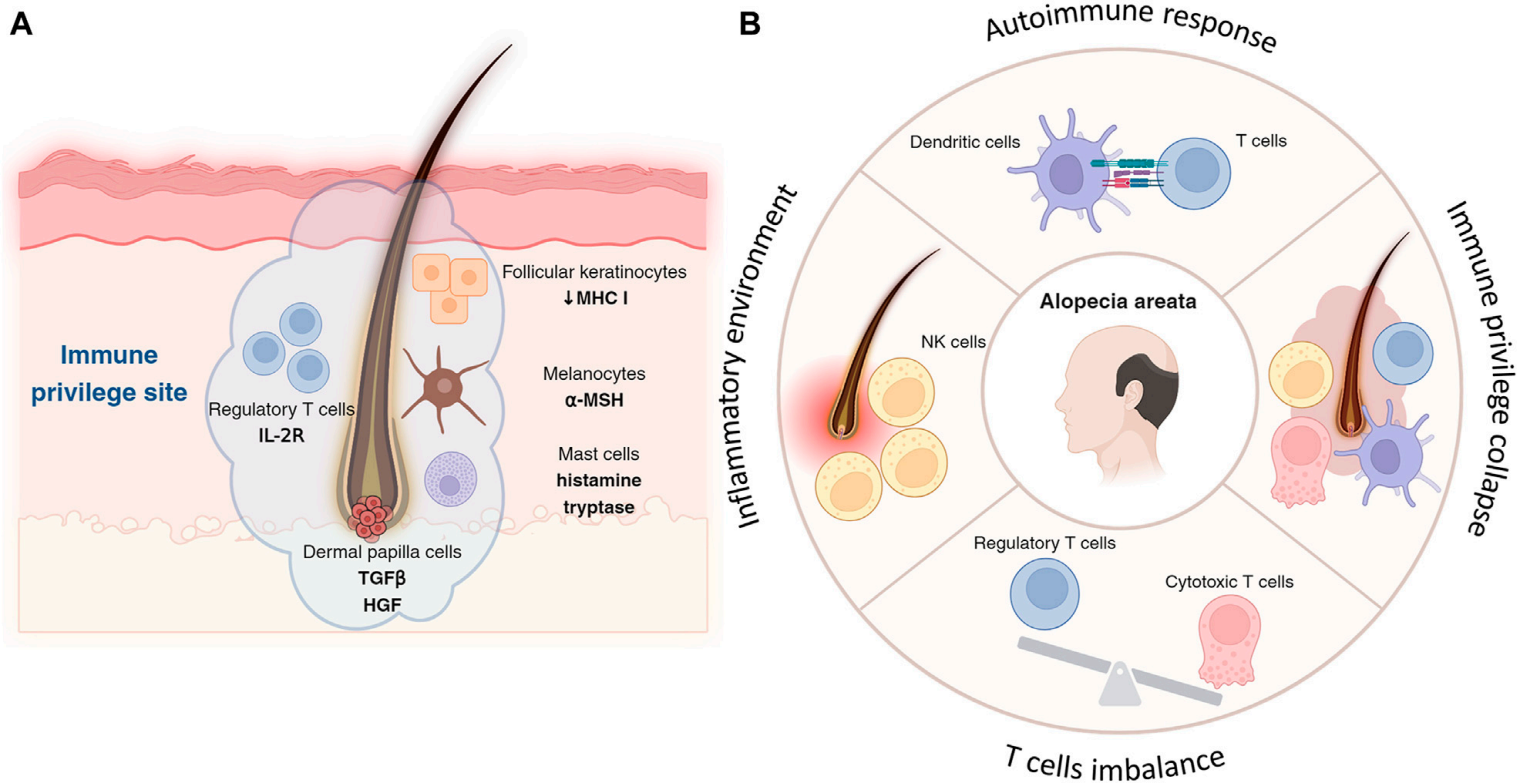
Immune privilege of hair follicle and its collapse. **(A)** Follicular keratinocytes, especially in the outer root sheath of hair follicles, express lower levels of MHC molecules, reducing their ability to initiate immune responses and contributing to the follicle’s immune privilege. Located in the hair bulb, melanocytes not only produce hair pigment but also secrete immunosuppressive factors, like α-MSH, aiding in immune privilege by suppressing inflammation. Concentrated around hair follicles more than in other skin areas, Treg cells, with their high-affinity IL-2 receptors, suppress immune responses to protect follicles from autoimmune attacks and inflammation. By releasing histamine and tryptase, mast cells in the hair follicle help maintain immune privilege, modulating immune responses and reducing inflammation to support a suppressive microenvironment. **(B)** Autoimmune assault is often initiated by cytotoxic T cells, which target and damage the hair follicles. T cells target hair follicles, leading to hair loss, with a notable imbalance between cytotoxic T cells and impaired or reduced Treg cells contributing to the disease progression. Key immune players including NK cells and dendritic cells exacerbate the condition by creating an inflammatory environment and presenting follicle antigens to T cells, respectively.

Another important feature of the hair follicle is immune privilege (Paus et al., 2003; Bertolini et al., 2020; Lee and Choi, 2024). The immune privilege is a set of mechanisms that prevent or suppress active cytotoxic attacks from immune cells to prevent potential damage of organs by inflammatory processes. This concept is crucial for maintaining tissue integrity in areas where inflammation can have detrimental effects. There are many immune-privileged sites in the body including the anterior chamber of the eye, blood-brain barrier, fetal trophoblast, testes, nervous system, and matrix under the proximal nail fold (Kanellopoulos-Langevin et al., 2003; Ito et al., 2005; Carson et al., 2006; Benhar et al., 2012; Li et al., 2012). The hair follicle also is characterized as a unique immune-privileged environment with diverse cellular components (Figure 2A).

### Follicular keratinocytes

Keratinocytes in the hair follicle, particularly those in the outer root sheath, contribute to the immune privilege by expressing lower levels of major histocompatibility complex (MHC) molecules, which are crucial for antigen presentation to T cells (Paus et al., 1998). This reduced expression limits the follicle’s ability to trigger an immune response.

### Melanocytes

Melanocytes in the hair bulb are responsible for pigment production in the hair and also play a role in immune regulation. Melanocytes in the hair follicle can produce immunosuppressive factors, such as alpha-melanocyte-stimulating hormone (α-MSH), which helps in maintaining the immune-privileged status by suppressing inflammation (Billingham and Silvers, 1971; Clemson et al., 2017).

### Treg cells

Treg cells help suppress immune responses and prevent autoimmunity, expressing high-affinity IL-2 receptors, which are crucial for their suppressive function, thereby protecting hair follicles from inflammation and autoimmune attacks (Cohen et al., 2024). Tregs are found in higher concentrations around hair follicles compared to other skin areas, contributing to the immunosuppressive microenvironment (Cohen et al., 2024).

### Mast cells

In the hair follicle, mast cells can contribute to immune privilege by releasing mediators such as histamine and tryptase, which modulate local immune responses and reduce inflammation around the hair follicles, suppress inflammation, and modulate the activity of other immune cells (Maurer et al., 1997; Bertolini et al., 2016).

### Dermal papilla cells

Dermal papilla works as a signaling center of hair follicle homeostasis by secreting several factors (Rahmani et al., 2014)*.* Among them, transforming growth factor-beta (TGF-β) and hepatocyte growth factor (HGF) impact not follicular keratinocytes as well as local immune cells. Therefore, these factors can contribute to the immunosuppressive microenvironment and help to maintain the immune privilege (Park et al., 2021).

### Endothelial cells

Endothelial cells express immunosuppressive molecules like indoleamine 2,3-dioxygenase (IDO). IDO recruits Treg cells near the hair follicles and fosters a microenvironment that limits inflammatory responses. This immunosuppressive network is essential for protecting the hair follicle from immune-mediated damage (Ito et al., 2008).

The sophisticated interplay of diverse cell types within the hair follicle microenvironment controls a unique immune privilege state for maintaining follicular integrity. Future researches that elucidate precise molecular mechanisms of sustaining the follicular immune privilege could unlock novel strategies for enhancing hair growth or preventing hair disorders.

## Abnormal immune niches in the alopecia

Abnormal immune niches can affect hair follicle homeostasis and regeneration. This leads to several skin disorders of hair growth, including alopecia areata that an autoimmune attack against hair follicles disrupts their normal growth cycle. Disruption of the balance between pro-inflammatory and anti-inflammatory signals can alter follicular immune privilege and cause hair loss eventually.

In alopecia, particularly in forms like alopecia areata, immune cells play a pivotal role in the pathogenesis of hair loss (Figure 2B). The condition is primarily characterized by an autoimmune response where T lymphocytes attack the hair follicles (McElwee et al., 2013). This autoimmune assault is often initiated by cytotoxic T cells, which target and damage the hair follicles, leading to hair loss. Treg cells, typically responsible for suppressing immune responses and maintaining tolerance, are found in reduced numbers or are functionally impaired in alopecia areata (Wan et al., 2023). The imbalance between effector T cells and Tregs is a critical factor in the progression of this disease. Another key player in alopecia areata is the natural killer (NK) cells, which are considered to contribute to the inflammatory environment around the hair follicles (Younes et al., 2022). Dendritic cells also play a role; they present antigens from the hair follicles to T cells, further propagating the autoimmune response (Abreu-Velez et al., 2009). The release of pro-inflammatory cytokines by these immune cells exacerbates the condition, leading to the disruption of the hair growth cycle (Liu et al., 2023), have shown promise in reversing hair loss in alopecia areata. Understanding the exact mechanisms of immune cell involvement in alopecia is crucial for developing more effective and targeted therapies for this condition.

The restoration of hair growth through immune modulation in alopecia areata represents a promising area of therapeutic research (Rahmani et al., 2020). Immune modulation aims to correct the dysregulated immune responses that target hair follicles, a key factor in alopecia pathogenesis. One of the most significant advancements in this field is the use of JAK inhibitors (Liu et al., 2023), which target specific pathways involved in the inflammatory process leading to hair loss. These inhibitors have shown efficacy in interrupting the signaling that causes the immune system to attack the hair follicles, thereby promoting hair regrowth. Another approach involves the use of corticosteroids (Fernando and Goldman, 2020), which suppress the overall immune response and reduce inflammation around the hair follicles. Topical immunotherapy, a method of inducing a mild allergic reaction, is believed to divert the immune system’s attention away from attacking the hair follicles (Singh and Lavanya, 2010). Drugs targeting the tumor necrosis factor (TNF) pathway, have been explored for their potential in modulating the immune response in alopecia (Gohary and Abdel Fattah, 2017). Cyclosporine, an immunosuppressant, has been used for its ability to inhibit T-cell activation, though its use is limited by side effects (Gohary and Abdel Fattah, 2017). Methotrexate, another systemic immunosuppressant, can reduce the heightened immune activity responsible for follicular damage (Hammerschmidt and Mulinari Brenner, 2014).

Ongoing research is focused on identifying specific immune cells and cytokines involved in alopecia to develop more targeted immune-modulating therapies, potentially offering more effective treatment options with fewer side effects. These approaches underscore the critical role of the immune system in hair growth and the potential of immunomodulatory therapies in restoring hair in alopecia patients.

## Conclusion

Despite the significant technological advancements achieved in recent years, largely attributable to the enhancement of omics technologies, such as genomics, transcriptomics, proteomics, and metabolomics, numerous questions regarding the cellular interactions that underpin the genesis, functioning, and diseases associated with hair follicles remain unresolved. These remaining questions underscore the complexity of hair follicle biology and highlight the need for further studies for advanced scientific techniques. Integrating data from single-cell RNA sequencing, spatial transcriptomics, and intravital imaging methods promises to shed light on these intricate cellular interactions at the single-cell level and offers new insights into hair follicle development, regeneration, and pathology. This comprehensive approach will be crucial for developing targeted therapeutics and interventions for hair follicle-related disorders.
